# Dental Students’ Ability to Detect Only-Enamel Proximal Caries on Bitewing Radiographs

**DOI:** 10.7759/cureus.31593

**Published:** 2022-11-16

**Authors:** Mohamed Samir A Elnawawy, Harshkant Gharote

**Affiliations:** 1 Operative Dentistry and Endodontics, Batterjee Medical College, Jeddah, SAU; 2 Oral Medicine Division, Department of Basic and Preventive Sciences, Batterjee Medical College for Science and Technology, Jeddah, SAU

**Keywords:** ability, bitewing radiograph, proximal caries, only-enamel caries, dental students

## Abstract

Introduction

The dental students are trained to identify the proximal carious lesion using clinical tools and radiography over three years of academic curriculum. During these years the students are expected to learn detection of incipient carious lesions and take appropriate preventive/restorative measure.

Method

This study aimed at evaluating the ability of the students and interns to detect only-enamel proximal caries on the five digital bitewing radiographs. The digital bitewing radiographs were having incipient carious lesions involving enamel on proximal surface of mandibular first molars.

Results

A total of 101 participants (M = 29, F = 72) from fifth (D5) and sixth (D6) years of dentistry evaluated the radiographs and the KR_20 _values for D5, D6, and total sample were 0.79, 0.64, and 0.41 respectively.

Conclusion

Overall students’ assessment of detection of incipient carious lesion was low. There is need to enhance clinical and radiological cognitive skills among students with emphasis on interdisciplinary learning objectives.

## Introduction

Dental caries is a multifaceted disease encountered by oral healthcare providers in routine clinical practice. This condition indicates loss of dental hard tissue structure due to bacterial invasion. However, being painless initially, patients generally visit the dentists with the complaint of food lodgment, dentinal hypersensitivity, or pulpal involvement with considerable tooth involvement. Therefore, early detection of initial demineralization poses a challenge for the dentist during routine dental checkups to restrict and manage caries involving only enamel [1-3].

Interproximal carious lesions initiate between the contacting proximal surfaces of two adjacent teeth. They first appear clinically as opaque regions and are caused by loss of enamel translucency at the outermost enamel between the contact area and the free gingival margin [4]. The posterior proximal caries are frequently difficult to identify on the radiograph because of large proximal surfaces and the thin mineral loss initially presented by lesions on these surfaces [5].

Diagnostic systems currently applicable for the identification of carious lesions include fiber optic transillumination, contrast dyes, and the combination of typical clinical and radiographic examinations. Bitewing radiography is one of the preferred modalities along with clinical examination for the detection of proximal and carious lesions on enamel and dentin. This technique also helps in estimating the depth and monitoring the behavior of cavities and is indispensable to the detection of incipient carious lesions located in the proximal surfaces [1,6].

During dental education, the students are educated to detect the proximal carious lesion using clinical tools and radiography. This training is typically distributed over three years of academics in the Operative Dentistry syllabus, starting from third-year training till the internship. During these years, the students are expected to learn the detection of incipient carious lesions and implement the preventive measures [7]. This study aimed at evaluating the ability of the final year students and interns to detect only-enamel proximal caries on the digital bitewing radiographs selected by the operative dentist.

## Materials and methods

The cross-sectional study was conducted among fifth- (D5) and sixth (D6)-year dental students of Batterjee Medical College, Jeddah, Saudi Arabia. The study protocol was approved by the Institutional Ethical Committee- Batterjee Medical College, Jeddah as per the norms of the country (Approval no. UB-RES-2020-0044). Five digital bitewing radiographs with only-enamel caries on the proximal surface of mandibular first molars were randomly selected by the specialist in Operative Dentistry. The retrieved radiographs were from the available database of the Sirona Sidexis digital imaging system exposed on the VarioDG intraoral x-ray machine (Sirona, Germany) available in the institution. The radiographs were previously made for the consented patients who visited for the dental checkup in the dental institution and did not involve any human subjects.

The selected five radiographs were labeled as bitewing1 through bitewing 5 (BW1, BW2, BW3, BW4, and BW5). A total of 101 students (D5-40 and D6-61) as observers were enrolled after taking written consent for the study. A single monitor was used for display of all the radiographs with fixed contrast and brightness to avoid bias. Students scored the presence or absence of proximal caries as 1 or 0 for presence or absence respectively for five proximal surface lesions. The raw data so obtained were entered into an Excel sheet for statistical analysis.

Statistical analysis: Student’s t-test was used to calculate the significance of age at p-value <0.05. Chi-square test was applied for the evaluation of bitewing radiographs by both the groups. Kuder-Richardson 20 (KR20) test was performed to confirm reliability in D5, D6, and the total sample size.

## Results

There were 101 participants (M = 29, F = 72) from the fifth (D5) and sixth (D6) years of the Dentistry Program. The mean age of the students in D5 and D6 groups was statistically significant (Table 1). Detection of proximal caries by both the groups of students was performed for each bitewing radiograph (BW1 to BW5). The Chi-square statistic proved the non-significant relationship between the radiographic examinations performed by D5 and D6 groups (P>0.05) (Table 2). KR20 scores were calculated for each group and the combined sample and found to be less than acceptable minimum KR20 value of 0.8 (Table 3).

**Table 1:** Mean age of the D5 and D6 students

Parameter	No. of participants (N = 101)	Age (Years) Mean ± SD	p	Inference
D5	40	20.37±0.24	<0.00001	Significant
D6	61	21.53±0.74		

**Table 2:** Evaluation of only-enamel caries in bitewing radiographs by D5 and D6 students *BW: bitewing

Parameter	BW^*^1	BW2	BW3	BW4	BW5	Total	Chi-square statistic	p	Inference
D5 (40)	30	29	36	31	33	159	2.3157	0.677908	Non-significant
D6 (61)	47	42	37	45	52	223			

**Table 3:** KR20 scores for D5, D6, and the total sample size

Parameter	D5 (N = 40)	D6 (N = 61)	Total participants (N = 101)
KR_20_	0.72	0.64	0.41

## Discussion

Early carious lesions are difficult to diagnose with visual tactile methods, especially in the proximal surfaces of posterior teeth. Over the last several years, bitewing radiographs are a primary diagnostic tool in proximal caries detection, nevertheless, there are several caries detection and visualization systems available. With limitations to conventional radiography, digital imaging has the upper hand with the availability of software tools [6]. In this way, the advances in digital imaging have enhanced accuracy in proximal caries detection. Further, it is included in the undergraduate curriculum during training in the operative/restorative dentistry syllabus. Training in early detection of enamel-only caries can be useful in retaining most of the tooth structure and minimizes the cycle of treatment and retreatment [8].

The present study was planned to evaluate the ability of undergraduate dental students to detect enamel-only proximal caries in posterior proximal surfaces. A total of 101 students evaluated five digital bitewing radiographs having only-enamel caries on the proximal surface of mandibular first molars. The mean ages of the students from the D5 and D6 groups were found to be statistically significant.

The student’s scores for the presence or absence of only-enamel caries were tested for reliability using the Kuder-Richardson test. The reliability for D5 and D6 students was found to be 0.79 and 0.64 respectively, while the overall reliability was 0.41. The present study showed high reliability for D5 students as compared to D6 students with low overall reliability. Yet, the interobserver statistics between the groups has not been significant. Our findings for the D5 group were corresponding with Abu El-Ela et al. [9] although their comparison was among two sets of observers. Further, one study demonstrated a lower specificity of bitewing radiographs in the detection of enamel proximal caries as compared to the lasers-based clinical methods in non-cavitated proximal surfaces [10].

Detection and diagnostics of proximal caries mostly rely upon bitewing radiography that can be performed by using a bitewing film holder or using disposable bitewing tabs. Therefore, most of the studies considered conventional and/or digital bitewings especially using general dentists or specialists as observers for the detection of proximal caries [11,12]. However, the present study planned to include undergraduate students as observers.

The findings of this study could be considered a first step in establishing certain criteria for vigorous training in radiographic interpretation during undergraduate training. A potential source of bias in assessing diagnostic accuracy among observers is the availability of reference standards and test methods. So, conducting the assessment at various time intervals to achieve higher inter- and intra-observer reliability is needed. Nevertheless, time limitations due to multiple interdisciplinary training can act as a constraint in conducting such studies among students [13].

The results of this study are directed at the development of course-learning objectives for oral radiology training, with an emphasis on interpretation from the Restorative Dentistry viewpoint. As a consequence, an initial phase of pedagogical plans and successive skills development can be accomplished from basic science training to clinical exercise during the progression of curriculum. In this sense, more case-based studies should be included during formal training to render higher cognitive skills in students [7,10].

The present study planned to evaluate the ability of undergraduate students to diagnose only-enamel proximal caries on digital bitewing radiographs. The results were inconsistent among students within and between the groups. Further, because of differences in the number of participants in the two groups, the reliability scores were not at the acceptable level. Constraints with the available sources and limited experience during their formative training are the causes of not achieving the desired goals in radiographic interpretation. However, limited availability of scientific literature in relation to the present study restricted the comparison of the data.

## Conclusions

Early diagnosis of only-enamel proximal caries plays a vital role in its management and prevention. The training of dental students during graduation definitely influences the overall ability in caries detection in private practice. The present study suggests a need for future research for formulation of interdisciplinary course-learning objectives between Oral Radiology and Restorative Dentistry. These objectives can improve the outcomes of caries detection ability. Further, blended learning with multidisciplinary approach can help plan an enriched curriculum for the undergraduate dental student.

The study also emphasizes the need for the comparative analysis of clinical and radiographic diagnostic skills among students for detection of various carious lesions. This would put forth the broader scope for understanding of cognitive domains in clinical learning process. Further, identifying statistical variables like specificity and sensitivity in detection of only-enamel caries can guide judiciously to take preventive measures.
